# Microfibrillar-Associated Protein 4: A Potential Biomarker for Screening for Liver Fibrosis in a Mixed Patient Cohort

**DOI:** 10.1371/journal.pone.0140418

**Published:** 2015-10-13

**Authors:** Susanne Gjørup Sækmose, Belinda Mössner, Peer Brehm Christensen, Kristoffer Lindvig, Anders Schlosser, René Holst, Torben Barington, Uffe Holmskov, Grith Lykke Sorensen

**Affiliations:** 1 Institute of Molecular Medicine, University of Southern Denmark, Odense, Denmark; 2 Department of Clinical Immunology, Naestved Hospital, Naestved, Denmark; 3 Department of Infectious Diseases, Odense University Hospital, Odense, Denmark; 4 Department of Biostatistics, Institute of Regional Health Research, University of Southern Denmark, Odense, Denmark; 5 Department of Clinical Immunology, Odense University Hospital, Odense, Denmark; 6 Odense Patient data Explorative Network (OPEN), Odense University Hospital, Odense, Denmark; University of Navarra School of Medicine and Center for Applied Medical Research (CIMA), SPAIN

## Abstract

**Background and Aims:**

A method for assessment of liver fibrosis and cirrhosis without the need for a liver biopsy is desirable. Microfibrillar-associated protein 4 (MFAP4) is a suggested biomarker for identification of high-risk patients with severe fibrosis stages. This study aimed to examine associations between plasma MFAP4 (pMFAP4) and transient elastography or chronic hepatitis C virus infection in drug users and in a mixed patient cohort with increased risk of liver disease. Moreover, the study aimed to identify comorbidities that significantly influence pMFAP4.

**Methods:**

pMFAP4 was measured in samples from 351 drug users attending treatment centres and from 248 acutely hospitalized medical patients with mixed diagnoses. Linear and logistic multivariate regression analyses were performed and nonparametric receiver operating characteristic-curves for cirrhosis were used to estimate cut-off points for pMFAP4. Univariate and subgroup analyses were performed using non-parametric methods.

**Results:**

pMFAP4 increased significantly with liver fibrosis score. pMFAP4 was significantly associated with chronic viral infection in the drug users and with transient elastography in both cohorts. In the mixed patient cohort, pMFAP4 was significantly increased among patients with a previous diagnosis of liver disease or congestive heart failure compared to patients with other diagnoses.

**Conclusions:**

pMFAP4 has the potential to be used as an outreach-screening tool for liver fibrosis in drug users and in mixed medical patients. pMFAP4 level is positively associated with transient elastography, but additional studies are warranted to validate the possible use of pMFAP4 in larger cohorts and in combination with transient elastography.

## Introduction

Chronic liver disease resulting in fibrosis and cirrhosis is a significant cause of morbidity and mortality in a large number of patients worldwide [1], and more than half of the cases are caused by hepatitis B virus (HBV) and hepatitis C virus (HCV) infection. Recent studies estimate that >185 million people are positive for anti-HCV [2], and 240 million are HBsAg-positive [3]. Other causes of chronic liver disease include alcohol abuse, steatosis, insulin resistance and autoimmune diseases [4]. Treatment decisions are based on the degree of liver fibrosis or cirrhosis in patients with chronic HCV infection, and studies have reported cirrhosis development in 10–20% of HCV-positive drug users in later life [5,6].

In the management of patients suffering from chronic liver disease, diagnosis and staging of fibrosis is essential. However, the traditional assessment of fibrosis by liver biopsy has a number of disadvantages related to safety, cost and accessibility. Therefore, many studies have aimed to evaluate blood-based biomarkers, scanning methods or combinations thereof.

Transient elastography (TE) is a non-invasive tool for measuring liver stiffness as a surrogate of liver fibrosis, as liver stiffness increases due to changes in the microstructure when the deposition of extracellular matrix (ECM) increases [7]. This method is widely used due to its high accuracy in the diagnosis of advanced fibrosis, and recent studies have shown an association between liver stiffness and survival [8]. It is further suggested that combining liver stiffness measurements with serological markers of fibrosis may enhance the performance of non-invasive fibrosis testing [9,10].

Microfibrillar-associated protein 4 (MFAP4) is localized to extracellular matrix fibers, including elastin and collagen [11,12]. Both MFAP4 and its bovine homologue have been detected in a variety of tissues [13,14]. The MFAP4 protein is a disulfide-linked dimer that forms higher oligomeric structures [12] and has an N-terminal Arg-Gly-Asp (RGD) integrin binding sequence [15]. The biological function of MFAP4 remains largely unknown. A role in elastogenesis is suggested, although not demonstrated *in vivo* [16–19]. Moreover, MFAP4 is an integrin ligand capable of activating smooth muscle cells *in vitro* and *in vivo* [20]. Systemic MFAP4 has further been reported to be moderately depressed in patients with stable atherosclerosis [14], and a moderate association with chronic obstructive lung disease has been proposed [21].

A previous search for novel biomarkers in HCV-associated hepatic cirrhosis revealed MFAP4 expression to be upregulated in fibrotic septae [22]. Furthermore, systemic MFAP4 was demonstrated to increase significantly with progressive fibrosis stage, indicating that MFAP4 may be a novel candidate for a systemic biomarker. High diagnostic accuracy for the prediction of non-diseased liver compared to cirrhosis was found [22].

In the present study, we set out to investigate associations between plasma MFAP4 (pMFAP4) and transient elastography in a cohort of drug users. Using data from a population of acutely hospitalized, medical patients a secondary aim was to investigate how pMFAP4 levels varied with possible comorbidities.

## Methods

### Study populations

#### Drug user (DU) cohort

Plasma EDTA-samples were obtained from a population of drug users recruited while attending the drug treatment centres on the Danish island Funen (approximately 500,000 inhabitants) during a 4-month period, as previously described[23]. Enrolled individuals completed a questionnaire, underwent liver stiffness measurement by TE and underwent clinical examination for cirrhosis stigmata within ± 30 days from the time of blood sampling. Patients with a TE measurement ≥8 kPa were evaluated for further referral, though all patients with a TE measurement ≥12 kPa were referred for further evaluation of cirrhosis by liver biopsy. There were 351 patient samples available for pMFAP4 measurements; 28 patients underwent liver biopsy. All biopsies were scored according to the Metavir classification; the pathologists scoring the biopsies were blinded to results from the TE measurements.

#### Mixed patient cohort

The mixed patient cohort included acute medical patients admitted to the medical admissions ward at Odense University Hospital. Recruitment was performed as previously described[24]. Plasma EDTA samples were drawn, liver stiffness measurement by TE was performed, and a questionnaire focused on signs of liver disease and congestive heart failure (CHF) was completed. Discharge diagnosis and vital status after 30 days were extracted from the civil register, while causes of death were extracted from hospital records and the national register of death certificates. From hospital discharge registers all hospital admissions and discharge diagnoses from 1994 to the end of follow-up (April 2009) were extracted to classify patients according to the Charlson comorbidity index [25]. There were 248 patient samples available for pMFAP4 measurements.

### Ethical approvals

The study was conducted in accord with the Declaration of Helsinki. The DU cohort was registered with the Danish Data Protection Agency and the study was approved by the Regional Scientific Ethical Committee for Southern Denmark (Project-ID S-VF 20060078). The mixed patient cohort was registered with the Danish Data Protection Agency and the study was approved by the Regional Scientific Ethical Committee for Southern Denmark (Project-ID: 2008-41-2449 and S-20080101). Written informed consent was obtained from all patients.

### Measurement of pMFAP4 concentrations by enzyme-linked immunosorbent assay (ELISA)

The ELISA setup was similar to that previously described [26]. Briefly, a sandwich ELISA was performed with two different monoclonal anti-MFAP4 IgGs (HG-HYB 7–5 and biotinylated HG-HYB 7–18) and detection by streptavidin-conjugated horseradish peroxidase was followed by color development by OPD turnover that was attenuated by H_2_SO_4_. A calibration curve was obtained through the use of a recombinant protein representing full-length human MFAP4 expressed in Flp-In^TM^ T-REx^TM^ CHO cells as previously described [12]. Standard two-fold dilution calibration-curve samples, quality control samples, and patient samples were analysed in duplicate.

### Statistical methods

Univariate associations between pMFAP4 and each potential covariate were calculated using Kendall’s rank tests. Multivariate analysis was performed using linear regression with backward elimination of insignificant variables. Box-cox analysis and relevant transformation to approach normal distribution was performed. Standard post-estimation analysis was performed to address problems concerning co-linearity. Subgroup analysis was performed using the Kruskal-Wallis equality-of-populations rank test and the Wilcoxon rank sum test. Comparative, nonparametric receiver operating characteristic analyses were performed for markers of liver fibrosis. Finally, multivariate logistic regression was performed to identify cut-off points for pMFAP4 using receiver operating characteristic curves for cirrhosis (Metavir fibrosis score F4). P-values below 0.05 were considered significant, and tests were two-sided when relevant. All statistical analyses were performed using STATA version 11.2.

## Results

### pMFAP4 increases with fibrosis stage verified by liver biopsy

**Fig 1:**
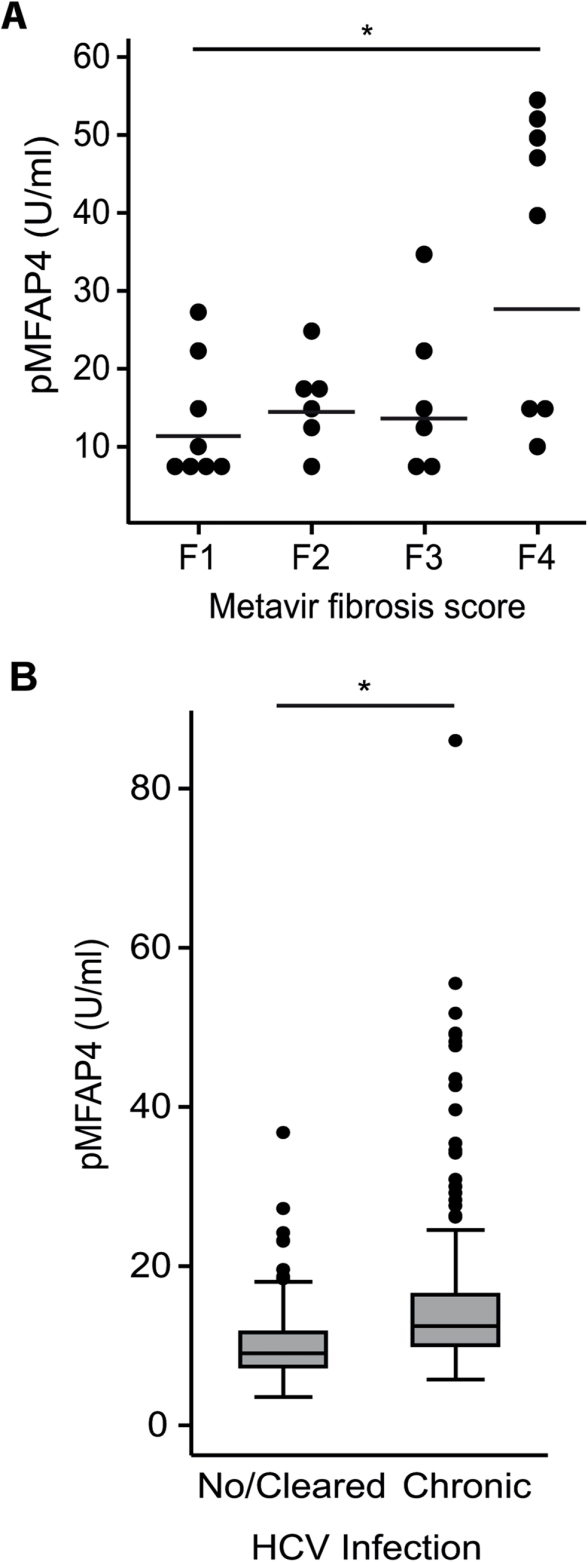
pMFAP4 levels stratified by Metavir fibrosis score (F1-F4) and HCV infection in the DU cohort. A. F1 denotes portal fibrosis, F2 denotes septal fibrosis (few), F3 denotes septal fibrosis (many), and F4 denotes cirrhosis. No biopsies were classified as F0 (no fibrosis). The horizontal lines represent mean pMFAP4 level within each Metavir fibrosis group; each dot represents individual pMFAP4 measurements. B. The boxes illustrate the 25^th^, 50^th^ and 75^th^ percentiles of pMFAP4 measurements in patients with no or cleared HCV infection and with chronic HCV infection. The whiskers illustrate ±1.5 inter quartile ranges. * Significant difference determined by the Wilcoxon rank sum test, p<0.005.

Demographic and biochemical parameters have been previously described [23,24]. The basic characteristics and mean pMFAP4 from the DU cohort and from the mixed patient cohort are presented in Table 1. Forty-five patients with liver stiffness measurement >12 kPa in the DU cohort were referred to a liver biopsy and 28 (62%) were accepted for analysis. The association of the Metavir fibrosis score with the measured pMFAP4 levels is illustrated in Fig 1A. The increase in pMFAP4 in F4 relative to F0 was significant (p = 0.02). Analysis of a threshold value of pMFAP4 to identify patients with confirmed cirrhosis showed that a cut-off level of 20 U/ml yielded a sensitivity of 62.5% and a specificity of 75% (Table 2). The specificity was increased to 85% by increasing the cut-off to 24 U/ml.

**Table 1:** pMFAP4 in the patient populations.

	Drug user cohort (n = 351)	Mixed patient cohort (n = 248)
*Gender*		
Male, n (%)	255 (73%)	115 (46%)
Female, n (%)	96 (27%)	133 (54%)
*Age*		
Range, years	21–68	17–97
Mean, years ± SD	41.3 ± 8.8	65.4 ± 19.6
*pMFAP4*		
Range, U/ml	3.6–86.0	3.2–106.2
Median (male/female)	10.3 (10.1/10.8)	12.2 (10.8/13.1)
Mean, U/ml (95% CI)	10.7 (10.2–11.2)	12.7 (11.7–13.9)

SD = standard deviation. Mean and 95% confidence intervals are calculated using transformed values for pMFAP4 and relevant back-transformation, theta = -0.2.

**Table 2:** Sensitivity and specificity for detecting cirrhosis (Metavir F4) with different cut-off levels for pMFAP4.

Cut-off	Sensitivity (%)	Specificity (%)	Correctly classified
≥ 8	100.0	25.0	46.4
≥ 12	87.5	45.0	57.1
≥ 16	62.5	65.0	64.3
≥ 20	62.5	75.0	71.4
≥ 24	62.5	85.0	78.6

The results are obtained using results from the 28 patients within the DU cohort with a liver biopsy. Area under the curve (AUROC): 0.76, 95% confidence interval 0.56–0.95.

### pMFAP4 increases with chronic HCV infection among drug users

Prevalence of HCV in the DU cohort was previously determined to be 42% [27]. All patients were additionally screened for HBV and human immunodeficiency virus (HIV); two patients were diagnosed with HIV, and three were found to suffer from chronic HBV. These patients were excluded from further subgroup analysis, and the remaining patients were divided into the following two groups: “no/cleared HCV infection” (HCV-RNA negative) and “chronic HCV infection” (HCV-RNA positive). The variations in individual pMFAP4 measurements in the two groups are illustrated in Fig 1B. The mean value of pMFAP4 in the no/cleared HCV infection group was found to be 9.2 U/ml (95% CI: 4.9–17.4) whereas the patients suffering from chronic HCV had a higher mean pMFAP4 level of 13.5 U/ml (95% CI: 6.7–47.6) (p<0.005).

### pMFAP4 significantly increases with TE

**Fig 2:**
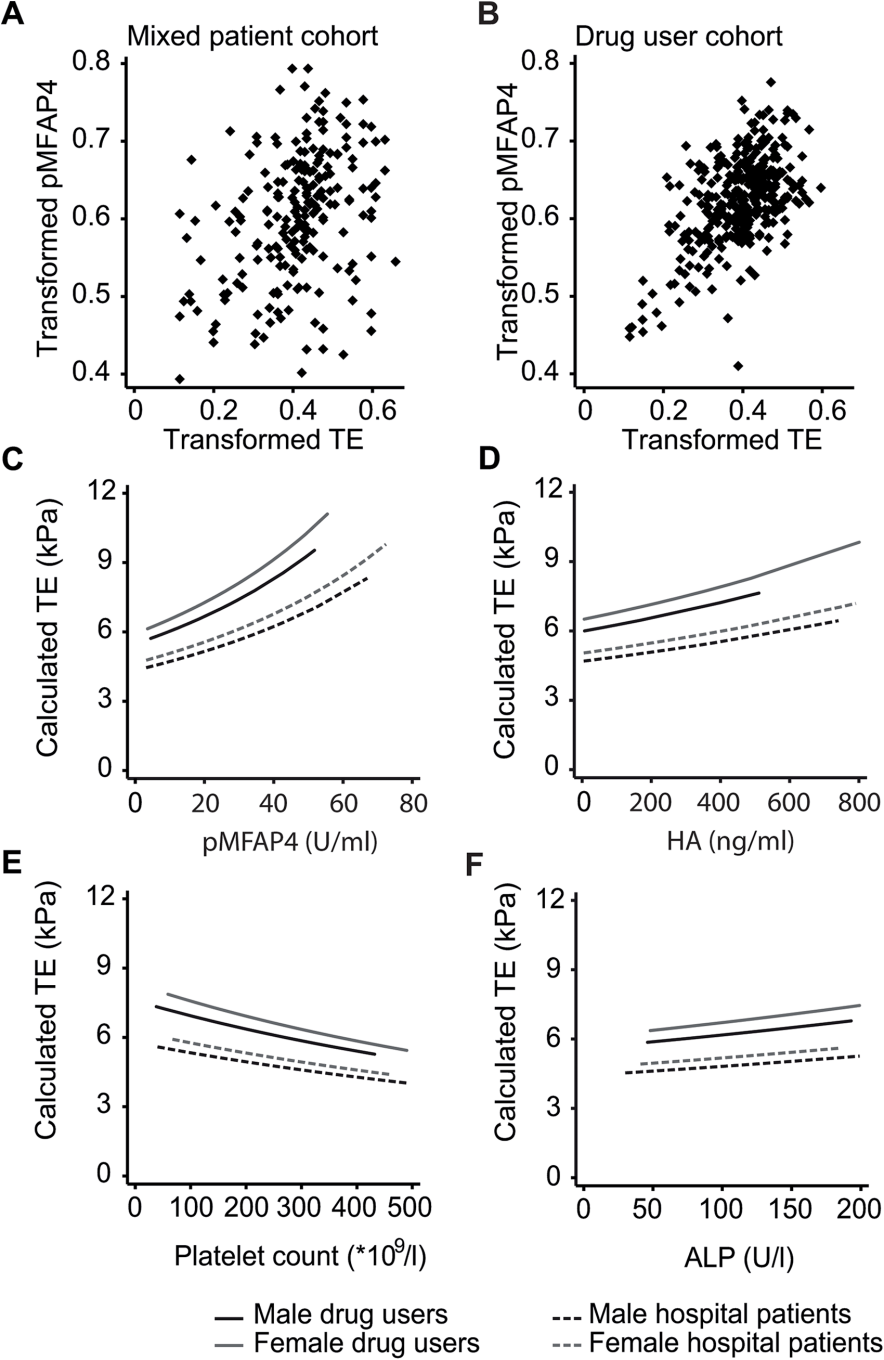
Significantly associated variables in the mixed patient cohort and the DU cohort. A. Correlation between pMFAP4 and TE in the mixed patient cohort using transformed data. B. Correlation within the DU cohort. Optimal transformations were determined using box-cox analysis. Univariate correlation was investigated by Kendall’s rank test and was significant for both populations (both: p<0.005). C. Variation of TE with pMFAP4 is calculated using median levels of age, HA, platelets, and ALP in the model. D. Variation of TE with HA is calculated using median levels of age, pMFAP4, platelets, and ALP in the model. E. Variation of TE with platelet count is calculated using median levels of age, pMFAP4, HA, and ALP in the model. F. Variation of TE with ALP is calculated using median levels of age, pMFAP4, HA, platelets, and ALP in the model.

Univariate correlation between pMFAP4 and TE was significant within both patient cohorts (DU cohort: p<0.005; mixed patient cohort: p<0.005) as illustrated in Fig 2A and 2B. TE was significantly associated with increasing pMFAP4, hyaluronic acid (HA), and alkaline phosphatase (ALP) in both cohorts, and it was also associated with decreasing platelet count and HCV-RNA positivity in the DU cohort (data not shown). When including both patient populations in the same regression model, TE was significantly associated with increasing pMFAP4, HA, ALP, and decreasing platelet count, as well as with female sex and increasing age (Fig 2D–2F). The *study cohort* was significantly associated with the outcome when included as a categorical variable. Post-estimation analysis showed no significant influence on the analysis from co-linearity between variables.

### pMFAP4 was increased among those in the mixed patient cohort with liver disease and CHF

**Fig 3:**
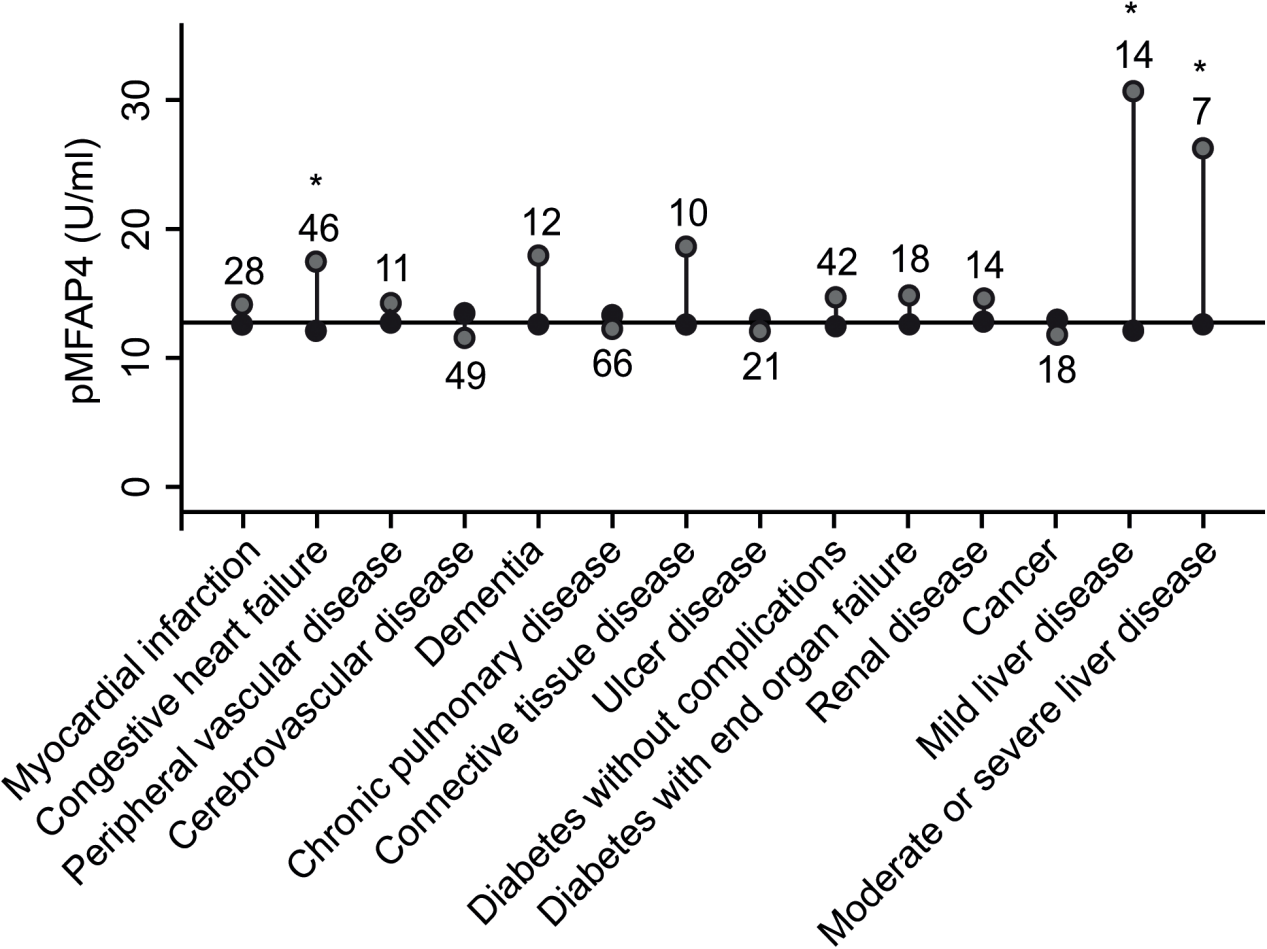
pMFAP4 level in the mixed patient cohort stratified by Charlson comorbidity group. The plot shows differences in mean pMFAP4 level between diseased and not-diseased patients according to Charlson comorbidity groups (not-diseased level calculated merging pMFAP4 measurements for all patients without the diagnosis in question). The black horizontal line represents the mean pMFAP4 for the whole population. Light gray circles illustrate the mean pMFAP4 value in the diagnosis group; the black circles illustrate the mean pMFAP4 of the remaining patients without diagnosis. The number of patients with the diagnosis in question is indicated. * Mean pMFAP4 significantly different from the rest of the cohort by the Wilcoxon rank sum test.

In the mixed patient cohort, discharge diagnoses from previous hospital admissions were used to classify the patients into 17 groups of medical conditions according to the Charlson comorbidity index [25]. The levels of pMFAP4 for each condition are shown in Fig 3. Both univariate analysis and a subsequent multiple linear regression demonstrated that the mean pMFAP4 was significantly higher in patients suffering from CHF (p<0.005) and those with liver disease (mild/moderate/severe: p<0.005).

In the mixed patient cohort, 36 of 248 (14.5%) patients did not have a valid liver stiffness measurement; among patients with a history of liver disease or CHF, 14 of 60 (23.3%) patients did not have a valid liver stiffness measurement. Among the remaining 46 patients with a valid scan, none had a history of both liver disease and CHF. Median pMFAP4 was approximately 2-fold increased in patients with liver disease relative to patients with CHF, whereas TE was approximately 4-fold increased. No significant differences between the two subgroups were found for HA, ALP or platelet-count.

Among the 16 patients with a history of liver disease, 5 patients had an admittance diagnosis related to liver disease (cirrhosis hepatis, peritonitis, esophageal varices). Mean pMFAP4 in these 5 patients (55.2 U/ ml, 95% CI: 16.7–93.6) was non-significantly increased relative to pMFAP4 measured in patients exclusively with a historical liver diagnoses (31.7 U/ml, 95% CI: 17.1–46.3) (p = 0.1).

No patients from the mixed patient cohort had a liver biopsy performed. Therefore, to evaluate the association between pMFAP4 and cirrhosis, a TE measurement above 17 kPA was used to theoretically differentiate cirrhotic from non-cirrhotic patients according to the limit proposed in a recent review of this issue [28]. This discrimination level suggested cirrhosis in 9 of 11 patients with known liver disease and in 5 of 35 patients with CHF.

## Discussion

In the present study, we confirmed that pMFAP4 increased with Metavir fibrosis score and that the pMFAP4 level was significantly higher among patients with chronic HCV infection. We found significantly increasing pMFAP4 levels with increasing TE in both the DU cohort and the mixed patient cohort, both when analyzed separately and together. Besides liver disease, only CHF appeared to increase pMFAP4 significantly among patients in the mixed patient cohort.

The physiological function of MFAP4 in liver disease is largely unknown. In the cirrhotic liver, hepatic stellate cells are thought to be the main cell type responsible for MFAP4 synthesis, as visualized by staining of the cirrhotic septa [22]. The level of MFAP4 is increased more than 40-fold in the cirrhotic septa, which is why a role in the remodelling process of the ECM in fibrogenesis has been hypothesized. Furthermore, serum MFAP4 level was found to increase significantly with progressive fibrosis stage among patients suffering from HCV infection [22]; on the contrary, systemic MFAP4 level shows a limited basal variation in healthy individuals [26]. These findings prompted us to investigate the biomarker potential of pMFAP4 in comparison to TE in a cohort of drug users, which is a population known to have an increased prevalence of HCV infection and thus an increased risk of liver fibrosis.

In the drug user cohort, a significant association between pMFAP4 and TE was found. This association was consistent when other markers were included in the analysis. Likewise, a significant association between pMFAP4 and TE was found in a separate mixed patient cohort. When the cohorts were analysed together, the significant association between pMFAP4 and TE remained, as did associations with HA, ALP, platelet count, sex, and age.

TE is a non-invasive, easily performed ultra-sound technique for measuring liver fibrosis; it has been shown to be a clinically useful method to detect significant fibrosis, severe fibrosis and cirrhosis [29]. It is a promising technique for use in different types of liver diseases; however, certain factors can limit the reliability of the measurements, including steatosis, increased body mass index, ascites, and cholestasis. Moreover, the ability of TE to discriminate between stages II-IV of fibrosis is reduced [28] and additional non-invasive tools for liver fibrosis assessment are warranted.

In the subgroup of drug users who underwent a liver biopsy, a significant association between pMFAP4 and the Metavir fibrosis score was found, which agrees with previous findings [22]. Moreover, significantly increased levels of pMFAP4 were observed in patients with chronic HCV infection in the DU cohort. In the previous study by Mölleken et al.[22], a serum MFAP4 concentration of 24 U/ml was suggested as the decision threshold for the detection of cirrhosis. This estimate is not directly comparable to the cut-off levels for the level of MFAP4 when measured in plasma [26]. Here, a putative cut-off level for pMFAP4 of 20 U/ml correctly classified 71% of the cirrhosis patients with a specificity of 75% and a sensitivity of 62.5%. This estimate is of course limited by the low number of biopsies in the DU cohort.

Further limitations included that the compliance is low in a drug user population and that it is difficult to obtain samples from the same individual at several, fixed time-points. Therefore, each individual was only sampled once and the use of pMFAP4 to assess progression of liver damage over time could not be assessed. Longitudinal studies in more compliant patient populations are needed to address this issue.

In the mixed patient cohort, a significantly elevated level of pMFAP4 was only found among patients with liver disease or CHF. The pMFAP4 level was significantly higher in the liver disease patients relative to the CHF patients. The moderately elevated levels of pMFAP4 within the group of CHF patients may result from fibrotic processes in the heart, or they may be a result of backward failure causing congestion of the liver [30]. Some diseases are previously reported with potential to affect pMFAP4 including atherosclerosis [14] and chronic obstructive lung disease [21]. However, the observed effects were of limited magnitude relative to the changes observed in systemic MFAP4 in liver fibrosis and in the present study pMFAP4 variation was not significantly affected by such diagnoses.

A limitation for the mixed patient cohort study was the low number of patients within certain comorbidity groups, and also that many patients were admitted to the hospital due to an acute illness while suffering from one or several chronic diseases. The diversity of admittance diagnoses limited the ability to clarify if acute events independently influences systemic MFAP4. Our data suggested that acute events in liver disease may increase pMFAP4, but this aspect should optimally to be tested in larger studies and in studies of disease progression. However, pMFAP4 was most significantly increased in patients with liver disease and to a lesser degree CHF among patients with a wide range of acute and chronic diseases supporting that pMFAP4 predominantly is increased by liver disease. Previous studies did not show any induction in systemic MFAP4 during acute events in chronic obstructive lung disease [21] or cardiovascular disease [14].

Overall, the present study confirmed that pMFAP4 levels are markedly induced in liver fibrosis, which suggests applicability of MFAP4 as a novel non-invasive biomarker for assessment of hepatic fibrosis and identification of high-risk patients with severe fibrosis stages Larger studies are warranted for the validation hereof. The combination of MFAP4 measurements with existing tests might lead to a more accurate non-invasive diagnosis of hepatic fibrosis. Measurements of other new potential biomarkers for staging liver fibrosis such as fibulin-5, MMP-2 or TIMP-1 [31,32] further have the potential to improve the accuracy of non-invasive estimates of liver fibrosis.

In summary, this study demonstrates a significant association between pMFAP4 measurements and transient elastography. In drug users, the pMFAP4 level was significantly elevated in patients with chronic HCV infection, and in a mixed patient cohort, pMFAP4 was significantly elevated only among patients with a previous diagnosis of liver disease or CHF. Together, these observations support a biomarker potential of pMFAP4 in non-invasive screening for liver cirrhosis.
